# Effect of Ionomer–Solvent Interactions in PFSA Dispersions: Dispersion Morphology

**DOI:** 10.1021/acs.macromol.5c00613

**Published:** 2025-08-06

**Authors:** Melissa Novy, Denis Duchesne, Gregg Dahlke, Lisa P. Chen, Robert B. Moore

**Affiliations:** † Macromolecules Innovation Institute, Department of Chemistry, Virginia Tech, Blacksburg, Virginia 24061, United States; ‡ 5158 3M Advanced Materials Division, 3M Center, Building 280-1W-03, St. Paul, Minnesota 55144, United States

## Abstract

Alcohol–water solvent systems are commonly used to disperse perfluorosulfonic acid ionomers (PFSAs) for the fabrication of proton-exchange membranes, catalyst layers, and thin films. The profound effect of PFSA chemical structure, concentration, and solvent composition on the colloidal morphology of PFSA dispersions is investigated using small-angle X-ray scattering (SAXS). Five different PFSA chemical structures and three different binary alcohol–water solvent systems are utilized for relevance to industrial processing parameters. Although evidence for a cylindrical PFSA aggregate morphology is shown, the strong scattering maximum frequently observed in scattering patterns of semidilute PFSA dispersions is demonstrated to prevent the quantification of aggregate length. A semiempirical small-angle scattering model is introduced to fit the dispersion scattering patterns over a wide *q*-range and quantify aggregate dimensions on length scales smaller than the average interaggregate spacing. A thermodynamic model based on the self-assembly of cylindrical micelles is shown to describe aggregate dimensions. The surface area per side chain, σ, calculated from this model is observed to increase with increasing alcohol concentration in the solvent, while the aggregate radius and average number of chains per aggregate both decrease. These observations suggest that increases in σ may represent an increase in the hydrophobic component of the PFSA aggregates to the solvent due to improved compatibility between the PFSA and solvent. The PFSA–solvent interactions are studied in more detail in a second publication of the present study.

## Graphical abstract

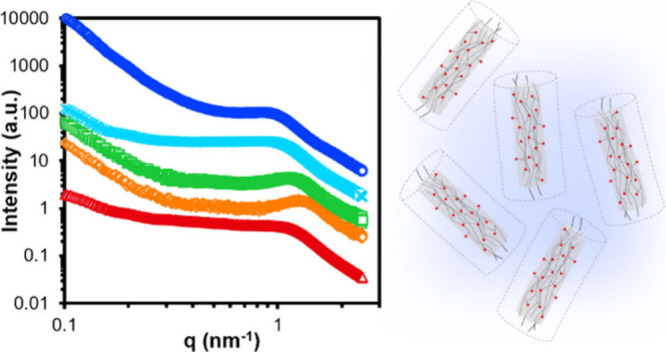

## Introduction

Perfluorosulfonic acid (PFSA) ionomers are widely used as solid electrolyte membranes and thin films in fuel cells, electrolyzers, redox flow batteries, sensors, and other applications requiring selective ion transport and good chemical and thermomechanical stability.^1,2^ PFSAs are copolymers of tetrafluoroethylene (TFE) and a perfluorosulfonic acid vinyl ether. As shown in Figure 1, the perfluorosulfonic acid component constitutes the hydrophilic side chains, while the perfluorinated hydrophobic backbone exists as segments of poly­(tetrafluoroethylene). The average number of TFE repeat units between side chains is represented by *n*. Summarized in Table 1, the three commercially available PFSA chemical structures are primarily differentiated from each other by (1) side chain length, designated by C2 (Aquivion, produced by Syensqo), C4 (produced by 3M Company), and long side chain or LSC (Nafion, produced by Chemours, and FORBLUE i-Series and S-Series, produced by AGC Inc.), and (2) side chain content, which is inversely proportional to equivalent weight (EW). EW is defined as grams of polymer per moles of SO_3_H and is equal to *n*MW_TFE_ + MW_sc_, where MW_TFE_ is the molar mass of the TFE repeat unit (100 g/mol), MW_sc_ is the molar mass of the side chain repeat unit (between ∼280 and 440 g/mol), and *n* is the average number of TFE repeat units between side chains.

**Figure 1:**
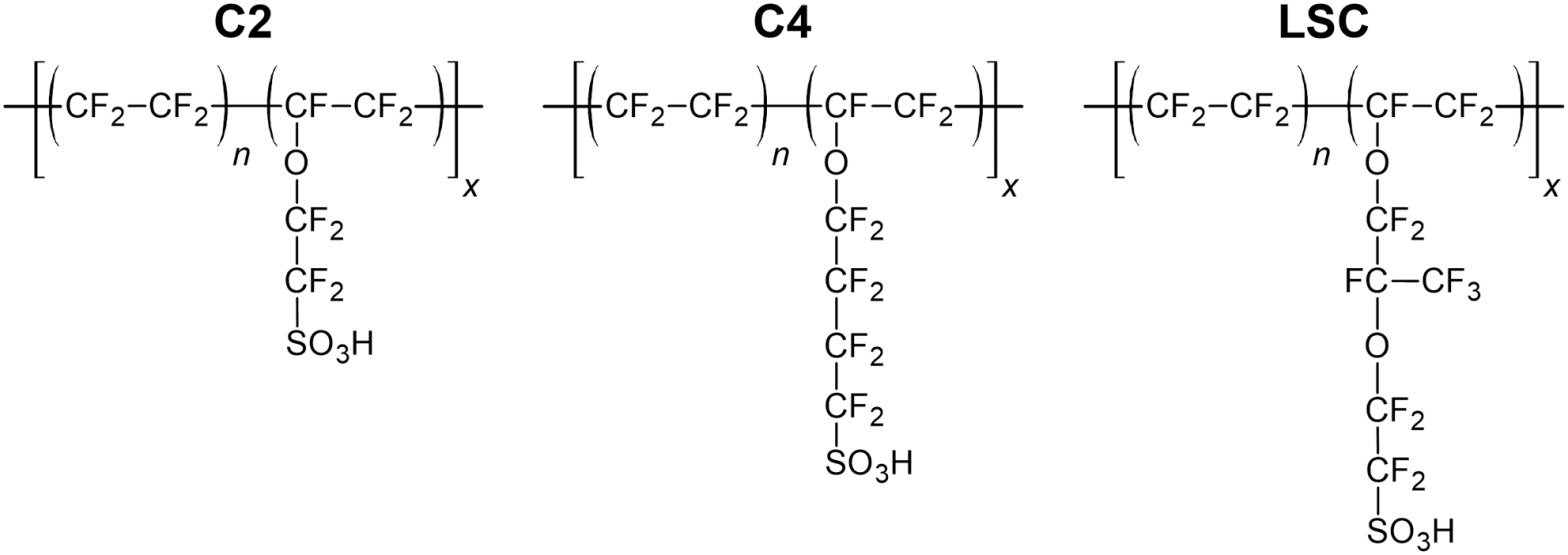
General chemical structures of the C2, C4, and LSC ionomers used in the present study.

**Table 1:** Side Chain and Backbone Composition Associated with the PFSA Ionomers Shown in Figure 1

Side Chain Structure	Nominal Equivalent Weight	Side Chain (mol %)	Backbone (mol %)	*n*
C2	830	15	85	5.5
C4	725	22	78	3.5
C4	790	19	81	4.1
C4	910	16	84	5.3
LSC	940	17	83	5.0

Fabrication of PFSA membranes, thin films, and catalyst layers is usually accomplished by first dispersing the solid ionomer in mixtures of alcohol and water or in high-boiling point solvents, followed by solvent evaporation at prescribed elevated temperature protocols.^3,4^ Investigations of dilute PFSA dispersions in protic solvents showed that PFSA does not completely dissolve in these solvent systems but forms colloidal dispersions.^1,5–7^ Many subsequent studies examined the colloidal PFSA morphology as a function of solvent composition to better understand its influence on the processing, morphology, and properties of PFSA membranes and thin films.^2,8–20^ Small-angle scattering (SAS),^5–7,21^ cryo-transmission electron microscopy (cryo-TEM),^22,23^ and electron spin resonance studies^24^ of dilute PFSA dispersions in binary alcohol–water solvent systems consequently established that PFSA aggregates consist of rodlike aggregates of chains, where the hydrophobic perfluorinated backbone forms the aggregate core and the hydrophilic sulfonic acid groups are located at the solvent–aggregate interface. Other aggregate morphologies, including random coils and gel-like particles, have been proposed for salt-neutralized PFSAs in aprotic solvents.^12,25^ Solvent properties including dielectric constant, interfacial energy, and solubility parameter^3,5,6,21,26–31^ were suggested to profoundly influence the form and dimensions of PFSA aggregates, leading to their implementation in solvent selection criteria for PFSA membrane, thin film, and catalyst ink fabrication.^8–10,13,14,20,29,32–34^

However, there are few studies to date of the morphology of PFSA dispersions conventionally used to cast freestanding membranes, i.e., nondilute PFSA dispersions in the acid form. The previous work on PFSA dispersions described in the paragraph above used either dilute and/or salt-neutralized PFSA dispersions to facilitate an analysis of the aggregate morphology. In addition, much of the current literature on PFSA dispersions pertains only to Nafion, a prototypical LSC ionomer. Excellent work by Gupit et al. studied the effect of PFSA concentration up to ∼30 wt % on aggregate morphology and rheology, but their investigation was limited to acid-form Nafion (1100 EW LSC) in a single ethanol–water solvent composition.^35^ More recent work by Wang et al. and Song et al. reported on the morphology of C2-type PFSA dispersions, but their studies used <10 wt % PFSA and just one solvent composition (ethanol–water).^22,23^ While Song et al. studied both C2-type and LSC-type PFSAs, only two C2 EWs and one LSC EW were used, providing only a partial investigation of the effect of EW and side chain length on aggregate morphology.^23^ The purpose of the present study is to systematically examine the relationship between nondilute PFSA dispersion morphology and solvent composition as a function of five different ionomer chemical structures (Figure 1 and Table 1) and three different binary alcohol–water solvent systems, using n-propanol (nPrOH), isopropanol (iPrOH), or ethanol (EtOH) as the alcohol. The ionomer concentrations (10 to 25 wt % PFSA) and solvent parameters used in this study have been selected primarily for relevance to the industrial fabrication of freestanding PFSA membranes. The present study demonstrates that SAS measurements of nondilute PFSA dispersions can provide quantifiable information on the average interaggregate spacing and radius of aggregates but no information on larger dimensions such as aggregate length. A SAS model is subsequently introduced to help quantify the morphology of the PFSA aggregates in dispersion and circumvent difficulties associated with fitting models to PFSA dispersion SAS patterns. For example, one issue is the presence of a scattering maximum in the SAS patterns due to interaggregate spatial correlations that increases in intensity with increasing PFSA concentration.^12,30,35,36^ Loppinet et al. reported that the scattering maximum is absent only at very low PFSA concentrations (<0.01 wt %) under salt-free conditions^30^ that are irrelevant to any current material applications of PFSAs. The present study will demonstrate that this scattering maximum obscures scattering associated with larger features of the aggregates in nondilute PFSA dispersions. Such difficulties include the quantification of aggregate length, where length values reported by previous PFSA dispersion SAS studies differ by up to 50%.^25,35,37^ Additionally, secondary aggregation has been suggested to affect aggregate length.^22^ SAS and dynamic light scattering (DLS) have been used to investigate the morphology of secondary aggregates (i.e., aggregates of aggregates) as a function of PFSA concentration. Interestingly, DLS measurements showed that the dimensions of secondary aggregates increased with increasing PFSA concentration,^38^ while SAS measurements indicated a decrease in dimensions with increasing concentration.^39^ Ultra-small-angle X-ray scattering was used to examine the effect of alcohol–water solvent composition on secondary aggregate dimensions. The findings suggested that the dimensions of secondary aggregates were larger in water-rich solvent systems, but there was no clear trend as a function of solvent composition.^40^ These studies show that the secondary PFSA aggregate morphology is difficult to quantify. The aggregate surface area per side chain derived from the thermodynamics of micellar self-assembly^41^ is proposed as an alternative to aggregate length and shown to depend on PFSA chemical structure and solvent composition. The application of micellar self-assembly concepts by the present study shows for the first time that aggregate morphology can be generalized across different PFSA chemical structures and a wide range of alcohol–water solvent compositions. The second part of this study further demonstrates a relationship between PFSA dispersion viscosity and the aggregate surface area per side chain obtained from the SAS model introduced in the present work. To the authors’ knowledge, this two-part study is the first to examine the relationship between PFSA dispersion morphology and viscosity for nondilute, acid-form dispersions for all three commercially available PFSA chemical structures in three different, commercially relevant alcohol–water solvent systems.

## Experimental Section

### Materials

Aquivion D83-24B (24 wt % 830 EW C2) aqueous dispersion was purchased from Millipore-Sigma. The water was evaporated from the dispersion at room temperature over several days to obtain the 830 EW C2 ionomer in solid form. The solid ionomer was then ground into a powder with a mortar and pestle. 3M Company provided PFSA powders of the C4 (at 725 EW, 790 EW, and 910 EW) and 940 EW LSC, and the sulfonyl fluoride nonionic precursor of 800 EW C4. Aquivion E87-05S (extruded 870 EW C2) membrane was purchased from FuelCellStore. All other reagents and solvents were purchased from Millipore-Sigma and used without further purification.

### Dispersion Preparation

PFSA powders are understood to be slightly hygroscopic. Thus, the moisture content of the powders was measured using a moisture analyzer by determining the mass loss after heating the powders at 100 °C for 1 h. The moisture content, M, was calculated using M = Δ*m*/*m*, where Δ*m* is the mass loss and *m* is the mass of the PFSA powder before heating. The moisture content was used to calculate the PFSA concentration and alcohol–water solvent composition of PFSA dispersions in eqs 1 through 3, where *f*_PFSA_, *f*_alcohol_, and *f*_H_2_O_ (*f*_PFSA_ + *f*_alcohol_ + *f*_H_2_O_ = 1) are the target weight fractions of the PFSA, alcohol, and water in the dispersion, respectively. The actual PFSA concentration by weight fraction in the dispersion is *C*_PFSA_, which includes an adjustment for the moisture content of the PFSA powder, M. The alcohol and water concentrations by weight fraction in the solvent alone are *c*_alcohol_ and *c*_water_, respectively (*c*_alcohol_ + *c*_water_ = 1). Throughout the present work, the reported alcohol and water concentrations refer to c_alcohol_ and c_water_.

1 $$C_{PFSA}=f_{PFSA}\left(1-M\right)$$

2 $$c_{alcohol}=\frac{f_{alcohol}}{1-f_{PFSA}\left(1-M\right)}$$

3 $$c_{water}=\frac{f_{water}+f_{PFSA}M}{1-f_{PFSA}\left(1-M\right)}$$

Nondilute dispersions (≥10 wt % PFSA) were prepared at room temperature by combining the solid PFSA and solvent mixture in a vial placed on a roller table for 24 to 48 h to disperse the PFSA. Dilute dispersions were prepared by diluting a 10 wt % PFSA dispersion with the same solvent composition and then mixing on a roller table for 24 h to redisperse the PFSA. All measurements were performed on the dispersions 2 days after preparation.

### Small-Angle X-ray Scattering Measurements

#### Static Measurements of ≤15 wt % PFSA Dispersions

Dispersions at PFSA concentrations ≤15 wt % and solvent blanks were sealed between two 25 μm-thick Kapton windows using silicone isolators (Grace Bio-Laboratories) with a path length of 0.26 cm. SAXS measurements were performed at beamline 12-ID-B at the Advanced Photon Source at Argonne National Laboratory (Lemont, Illinois, USA) under General User Proposal No. 68110. The high flux of the synchrotron X-ray source was used to improve the signal-to-noise ratio for relatively weakly scattering, relatively dilute dispersions,^42^ as scattered intensity decreases with decreasing PFSA concentration.^43^ The incident energy was 13.3 keV, corresponding to X-rays with a wavelength of 0.0932 nm. Two-dimensional SAXS patterns were obtained using a Pilatus 2M detector with a pixel size of 0.172 mm, exposure time of 0.3 s, and sample-to-detector distance of 1996 mm. The *q*-range was calibrated with a silver behenate standard. The SAXS patterns were corrected for sample thickness, transmission, background, and solvent.

#### Static Measurements of 25 wt % PFSA Dispersions

A custom wet cell was used to seal 25 wt % PFSA dispersions and solvent blanks between two 25 μm-thick Kapton windows with a path length of 0.1 cm. Static SAXS measurements were performed on a Rigaku S-Max 3000 3-pinhole SAXS system equipped with a rotating anode emitting X-rays with a wavelength of 0.154 nm (Cu K_α_). The sample-to-detector distance was 1005 mm, and the *q*-range was calibrated using a silver behenate standard. Two-dimensional SAXS patterns were obtained using a 2D multiwire, proportional counting, gas-filled detector, with an exposure time of 2 h. The SAXS patterns were corrected for sample thickness, transmission, background, and solvent and were put on an absolute scale by correction using a glassy carbon standard from the Advanced Photon Source.

Fitting of the SAXS patterns with SAS models was accomplished with Python in the Spyder integrated development environment or Sas View.

## Results and Discussion

The SAXS patterns of 25 wt % PFSA dispersions in mixed alcohol–water solvent are shown in Figure 2. The SAXS patterns are vertically offset for visualization, and the vertical shifting factors are summarized in Tables S1 and S2 in the Supporting Information. The low-*q* upturn at *q* < 0.3 nm^–1^ and scattering maximum near *q* = 0.9 to 1.3 nm^–1^ observed in the SAXS patterns in Figure 2 are common to ion-containing polymer solutions and dispersions. The low-*q* upturn has been observed in X-ray, neutron, and light scattering experiments in a variety of ion-containing polymer solutions or dispersions and is attributed to large-scale clusters of polymer chains.^44–47^ The scattering maximum, *q*_max_, is attributed to interparticle interferences and associated with an average spacing or distance between scattering particles.^48^ The presence of *q*_max_ is also indicative of spatial order among scattering particles on length scales around 2π/*q*_max_. Spatial order in ion-containing polymer solutions and dispersions is thought to arise from aggregation of polymer chains and/or electrostatic interactions between chains or aggregates, depending on the solvent and polymer ion content.^49^ In PFSA dispersions specifically, spatial order is attributed to primary hydrophobic aggregation of chains and secondary interaggregate electrostatic interactions.^6,7,21,25,50^ Thus, the *q*_max_ of the SAXS patterns in Figure 2 is representative of the average spacing between multichain aggregates in PFSA dispersions.^5–7,12,25,30,35,51,52^

**Figure 2:**
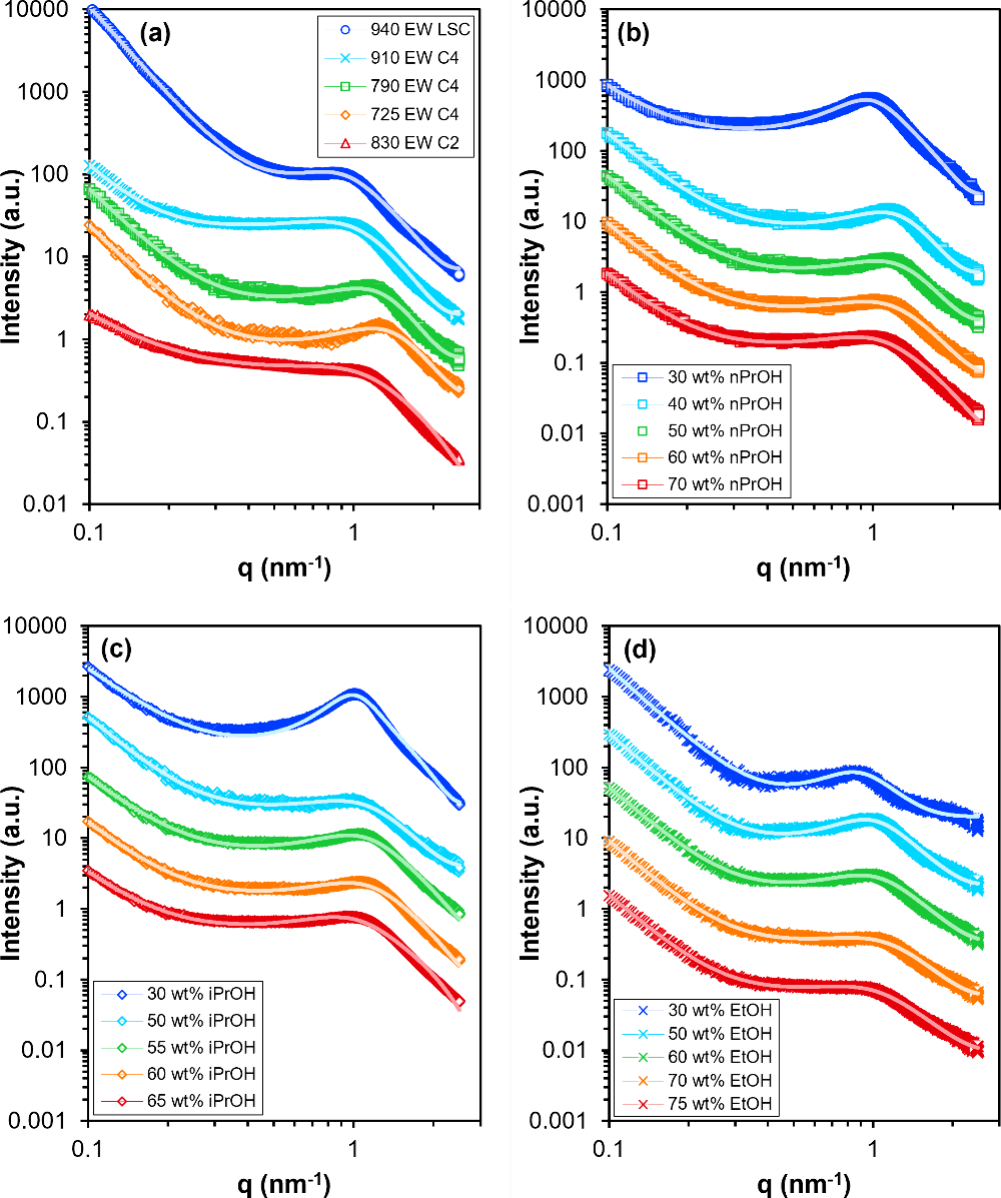
SAXS patterns of PFSA dispersions in alcohol–water solvent: (a) 25 wt % PFSA in 50 wt % nPrOH (balance water) as a function of PFSA chemical structure. (b–d) 25 wt % 790 EW C4 dispersions as a function of alcohol concentration in the solvent, with nPrOH, iPrOH, and EtOH as the alcohol, respectively. The SAXS patterns are shifted vertically for visualization. The solid lines are fits to the sum of a low-*q* power law and a cylindrical form factor with empirical structure factor.

The effect of PFSA chemical structure on dispersion morphology can be observed by comparing the SAXS patterns of five different PFSA ionomers at the same concentration and solvent composition, shown in Figure 2a. The value of *q*_max_ and the average interaggregate spacing equal to 2π/*q*_max_ are summarized in Table 2 as a function of PFSA chemical structure. The effect of side chain content on PFSA dispersion morphology can be examined by comparing the C4 ionomer series with the same side chain chemical structure but different EW: 725 EW C4, 790 EW C4, and 910 EW C4. The effect of side chain chemical structure can be examined by comparing the 830 EW C2, 910 EW C4, and 940 EW LSC dispersions, as these PFSAs have similar side chain contents (Table 1). The shaded box in Figure S1 in the Supporting Information demonstrates that, at a fixed side chain content, the PFSA EW increases as the side chain length increases. Table 2 shows that the average interaggregate spacing generally decreases as side chain length decreases or the side chain content increases. A decrease in average interaggregate spacing has been attributed to a decrease in aggregate radius (at constant PFSA concentration) by assuming a geometrically ordered spatial distribution of rodlike aggregates; i.e., the center-to-center spacing increases as the size of the scattering particle increases.^6,30^ Comparing the 830 EW C2, 910 EW C4, and 940 EW LSC PFSAs generally suggests that aggregates of shorter side chain PFSAs have smaller radii compared to longer side chain PFSAs. Comparing the EW series of C4 PFSAs generally suggests that higher side chain content (lower EW) PFSAs have smaller radii compared to lower side chain content (higher EW) PFSAs.

**Table 2:** Scattering Maximum, Average Interaggregate Distance, Aggregate Radius, Surface Area per Side Chain, and End-to-End Length of the Side Chain as a Function of PFSA Chemical Structure at 25 wt % PFSA in 50 wt % nPrOH (Balance Water)

PFSA Type	Scattering Maximum, *q*_max_ (nm^–1^)	Average Interaggregate Distance (nm)	Radius, *R* (nm)	Surface Area per Side Chain, σ (nm^2^)	Calculated End-to-End Side Chain Length, λ (nm)
830 EW C2	1.1	5.7	1.19 ± 0.04	1.10 ± 0.04	0.40 ± 0.01
725 EW C4	1.3	4.8	1.27 ± 0.06	0.90 ± 0.04	0.51 ± 0.02
790 EW C4	1.2	5.2	1.5 ± 0.1	0.86 ± 0.04	0.58 ± 0.03
910 EW C4	0.95	6.6	1.54 ± 0.07	0.94 ± 0.03	0.53 ± 0.02
940 EW LSC	0.93	6.8	1.52 ± 0.08	0.98 ± 0.05	0.51 ± 0.03

The effect of solvent composition on dispersion morphology was investigated for the 790 EW C4 ionomer by varying the concentration of alcohol, [alcohol], in the solvent from 30 to 75 wt % (balance water). The SAXS patterns of 25 wt % 790 EW C4 dispersions in different alcohol–water solvent compositions are shown in Figure 2b–d. To quantify the shape and position of *q*_max_, the SAXS patterns were fit with the sum of a low-*q* power law and the Teubner–Strey model, with fitting details provided in the Supporting Information in Tables S3 and S4 and eq S1. The Teubner–Strey model is widely used to model nondilute, two-phase systems of ion-containing polymers, colloids, and microemulsions, as it is capable of reproducing the characteristic small-angle scattering maximum observed in these systems.^53,54^ Furthermore, the Teubner–Strey model is a general model that does not account for the form or structure of scattering particles in these phase-separated systems. The relevant fitting parameters of the Teubner–Strey model are *q*_max_ and the correlation length, ξ. The parameter ξ in the Teubner–Strey model is inversely proportional to the width of the scattering maximum and thus is related to the distribution in interaggregate spacing. The correlation length may be interpreted as the degree of spatial order in the PFSA dispersions, where small correlation lengths are indicative of low spatial order.

Spatial ordering in PFSA dispersions is known to depend on solvent–ionomer interactions and interaggregate electrostatic interactions.^6,7,25,30^ A plot of the correlation length, ξ, as a function of [alcohol] shown in Figure 3 demonstrates that the correlation length increases as [alcohol] in the solvent decreases, consistent with a sharpening of *q*_max_ from a broad shoulder to a peak (Figure 2b–d). The increase in correlation length with decreasing [alcohol] suggests an increase in spatial order in the PFSA dispersions due to interactions between the solvent and ionomer. For example, the shape of *q*_max_ of sulfonated polystyrene and Nafion dispersions was found to change from a peak to a shoulder as the ratio of the components of the binary solvent system was changed to favor the relative solvation of the ionomer backbone over that of the ionic groups.^25,55^ A similar change in the shape of *q*_max_ from a peak to a shoulder with increasing [alcohol] can be observed for the 790 EW C4 ionomer dispersions in each of the three alcohol–water solvent systems (Figure 2b–d). In binary alcohol–water solvent systems, the alcohol is considered a better solvent for the backbone and water a better solvent for the hydrophilic side chains on a relative basis.^25,31,56,57^ Thus, the decrease in spatial order and broadening of *q*_max_ with increasing [alcohol] may be due to improved compatibility between the hydrophobic component of the PFSA aggregates and the solvent. A detailed analysis of PFSA–solvent interactions and interaggregate electrostatic interactions is given in a second part of this study in a separate work. The contribution of interaggregate electrostatic interactions to spatial ordering in PFSA dispersions is discussed later in the present work.

**Figure 3:**
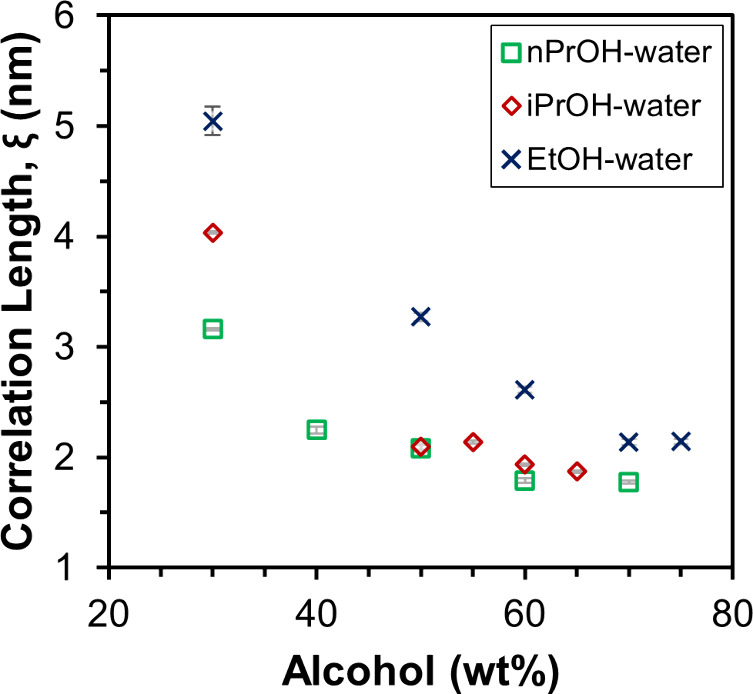
Correlation lengths of 25 wt % 790 EW C4 dispersions as a function of alcohol–water solvent composition, with nPrOH, iPrOH, or EtOH as the alcohol.

While general SAS models for colloidal systems, such as the Teubner–Strey model, can help to quantify the spatial order and interaggregate spacing of PFSA dispersions, more morphological detail can be obtained by investigating the form and structure of the aggregates. The total scattered intensity, *I*(*q*), of PFSA dispersions is described by *I*(*q*) = *A* × *P*(*q*) × *S*(*q*), where *q* is the scattering vector, *A* is a scaling factor, and *P*(*q*) is the form factor describing the shape and dimensions of the aggregates arising from intra-aggregate interactions. *S*(*q*) is the structure factor describing the interaggregate interactions and thus produces a scattering maximum in *I*(*q*) associated with *q*_max_. A variety of form factors have been applied to PFSA aggregates, including Debye chains (i.e., random coils), spheres, and cylinders with finite or infinite lengths.^7,12,21,25,35^ Structure factors used in the study of PFSA dispersions typically utilize either empirical methods^12,25,58^ or equations that describe the interaction potentials between charged scattering particles.^6,37^ However, no consensus on the morphology of PFSA aggregates in a given solvent system has been reached. For example, Welch et al. noted that SAS patterns of 2.5 wt % Na^+^-form Nafion in alcohol–water solvent could be fit equally well with a spherical form factor or cylindrical form factor with polydisperse radius.^25^ Additionally, SAS studies that used a cylindrical form factor to model PFSA aggregates have reported a range of cylinder lengths from 20 to over 35 nm, corresponding to a difference of over 50%.^35,37^

The difficulties of modeling PFSA dispersion SAS patterns with form and structure factors may be understood by first comparing the experimental data to different theoretical form factors. A spherical form factor and three different cylindrical form factors with fixed cylinder lengths of 10 nm, 30 nm and infinity are plotted along with an experimental 25 wt % PFSA dispersion SAXS pattern in Figure S2. Since the model form factors do not account for scattering due to interparticle interactions, the model form factors do not display the low-*q* upturn and scattering maximum present in the experimental SAXS pattern. Consequently, comparison of the form factors to the experimental SAXS pattern highlights the significant contribution of interaggregate correlations to the total scattered intensity over *q* < *q*_max_. Figure S2 also shows that the scattered intensity of the form factors differs more at lower values of *q*. The scattered intensity over *q* < *q*_max_ is observed to increase with increasing length of the form factor. However, any experimental scattered intensity at low *q* arising from the form of the PFSA aggregates is obscured by the low-*q* upturn and scattering maximum seen in the experimental SAXS pattern (Figure S2). Thus, scattering arising from interaggregate interactions in semidilute PFSA dispersions prevents the quantification of aggregate length in nondilute PFSA dispersions by SAS models.

Although the scattered intensity of PFSA dispersion SAS patterns at lower *q* is dominated by interaggregate interactions, the scattered intensity corresponding to *q* > *q*_max_ is unaffected by interaggregate correlations. Thus, the scattered intensity over *q* > *q*_max_ may be used to quantify characteristic dimensions of the aggregates on length scales <2π/*q*_max_,^59–62^ such as the aggregate radius. Accordingly, the experimental PFSA dispersion SAXS pattern shown in Figure S2 was fit over *q* > *q*_max_ with the four different form factors—spherical form factor and cylindrical form factor with fixed cylinder lengths of 10, 30, and ∞ nm—where the only independent fitting parameter was aggregate radius. The spherical and cylindrical form factor fitting equations are included in the Supporting Information in eqs S2, S3, and S4. The fitted radius values and the associated fitting uncertainties are listed in Table S5 as a function of form factor type—sphere or cylinder of a given length. Comparison of the fitted radius values for a given aggregate form factor shows that the radius is independent within error of the form factor type and/or cylinder length. This finding further confirms that only scattering corresponding to *q* > *q*_max_ can be used to obtain information on aggregate dimensions, while the scattered intensity over *q* < *q*_max_ is mainly affected by interaggregate correlations in nondilute PFSA dispersions.

Although aggregate form, i.e., spherical, cylindrical, elliptical, etc., cannot be accurately determined from SAS patterns of nondilute dispersions, SAS analyses may still be used to comment on aggregate morphology. Aldebert et al. found that the general form of aggregates in ionomer dispersions can be elucidated from the relationship between *q*_max_ and ionomer concentration.^6^ As an example, the SAXS patterns of 790 EW C4 dispersions in 50 wt % nPrOH (balance water) from 0.5 to 15 wt % PFSA are shown in Figure 4a. The low-*q* upturn at *q* < 0.2 nm^–1^ and scattering maximum between *q* = 0.1 to 1.5 nm^–1^ are observed in most scattering patterns. Note that the low-*q* upturn of the 0.5 wt % PFSA dispersion scattering pattern was difficult to resolve due to weak scattering from the dispersion, near that of the background scattering. As the PFSA concentration decreases, *q*_max_ is observed to shift to lower *q*, corresponding to an increase in average interaggregate distance (Figure 4a), as expected. A plot of *q*_max_ as a function of PFSA concentration, *C*_PFSA_, for dispersions of five different PFSA chemical structures from *C*_PFSA_ = 0.5 to 25 wt % is shown in Figure 4b. The scattering patterns of these PFSA dispersions are shown in Figure S3. The observed power law relationship between *q*_max_ and *C*_PFSA_ (i.e., scaling of *q*_max_ with *C*_PFSA_),^63^ with a scaling exponent between 0.4 and 0.5, is consistent with other SAS studies of PFSA dispersions.^5,7,30,35,50^ In these SAS studies, a scaling exponent of 0.5 was attributed to the dilution of aggregates with one dimension larger than the average interaggregate distance, indicating a rodlike aggregate morphology.^7,30,50^ Additionally, a scaling exponent of 0.5 has been well documented for ion-containing polymer solutions in the semidilute regime.^64,65^ Thus, the scaling behavior of *C*_PFSA_ with *q*_max_ of the PFSA dispersions shown in Figure 4b suggests the PFSA concentration range (0.5 wt % to 25 wt % PFSA) is within the semidilute regime and suggests the presence of rodlike aggregates for the five different PFSA chemical structures.

**Figure 4:**
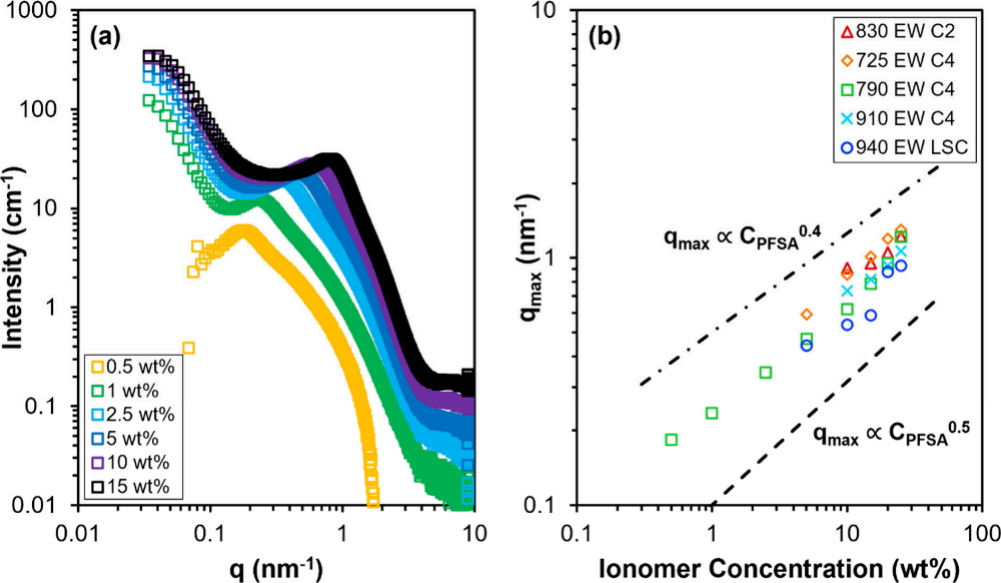
(a) Dispersion SAXS patterns of 790 EW C4 in 50 wt % nPrOH (balance water) as a function of PFSA concentration from 0.5 to 15 wt %. (b) Scaling of *q*_max_ with PFSA concentration, *C*_PFSA_, of 830 EW C2, 725 EW C4, 790 EW C4, 910 EW C4, and 940 EW LSC dispersions in 50 wt % nPrOH (balance water). The dashed lines correspond to scaling exponents of 0.4 and 0.5.

The analysis of SAXS patterns of semidilute PFSA dispersions in alcohol–water solvents by the present study has demonstrated that only aggregate radius values can be quantified and that the PFSA aggregates likely have a rodlike form factor. A SAS model was developed to account for these observations, enabling the SAXS patterns to be fit over the entire measured *q*-range and the quantification of aggregate radius and interaggregate correlations. Note that fitting SAS patterns over the full *q*-range of the measurement avoids the selection of an arbitrary *q*-range for fitting the scattering features of interest. The SAS model developed by the present study is described by eq 4 and is the sum of a low-*q* power law and the product of a cylindrical form factor, *P*_cyl_(*q*), and structure factor, *S*(*q*). The selection of a cylindrical form factor, *P*_cyl_(*q*), to model the shape of the PFSA aggregates is supported by SAS and simulation studies of PFSA dispersions in alcoholic solvents,^5,25,58,66^ as well as the data shown in Figure 4. The structure factor was developed by the present study to empirically represent the interaggregate interactions, thereby producing a peak in the model scattering pattern and fully modeling the SAXS patterns. The empirical structure factor is described by eq 5, where β is a constant associated with the scattered intensity as *q* approaches zero, *c*(2*qR*_ca_) is a function that represents hard sphere interparticle correlations,^67^ and *P*_cyl_(*q*) is the cylindrical form factor equal to eq S3. The radius of closest approach represents the assumption that aggregates cannot occupy the same space and are separated by some minimum distance, *R*_ca_. This empirical structure factor (eq 5) is derived from the Polymer Reference Interaction Site Model (PRISM) developed by Schweizer and Curro^68^ to describe the liquidlike structure and interparticle correlations of polymer solutions and melts.^69,70^ Such PRISM-based structure factors have also been used to model scattering from micellar solutions.^71–73^ The general form of a PRISM-based structure factor is $S\left(q\right)=\frac{1}{1-\beta c\left(q\right)P_{SC}\left(q\right)}$ , where the parameters β, *c*(*q*), and *P*_SC_(*q*) have similar meanings as described above. Notably, *P*_SC_(*q*) represents the form factor of a single chain within a micelle or polymer melt.^71^ However, application of the PRISM-based structure factor, in contrast to the empirical structure factor (eq 5), requires the mathematical coupling of β, *c*(*q*), and *P*_SC_(*q*): The constant β depends on *R*_ca_, particle length, and number density of the scattering particles, while *c*(*q*) and *P*_SC_(*q*) may both depend on *R*_ca_ and particle length.^71^

4 $$I\left(q\right)=\frac{A}{q^{D}}+P_{cyl}\left(q\right)S\left(q\right)$$

5 $$\begin{array}{l} S\left(q\right)=\frac{1}{1+\beta c\left(2qR_{ca}\right)P_{cyl}\left(q\right)}\\ \mathrm{where}\;c\left(2qR_{ca}\right)\approx 3\frac{\sin\left(2qR_{ca}\right)-\left[2qR_{ca}\;\cos\left(2qR_{ca}\right)\right]}{{\left(2qR_{ca}\right)}^{3}} \end{array}$$

If system parameters such as particle length and number density are well characterized, such as in biological systems,^71,73^ the coupling of β, *c*(*q*), and *P*_SC_(*q*) does not typically present issues in modeling SAS patterns with PRISM-based structure factors. However, the inability to quantify the length of PFSA aggregates by SAXS in the present study results in multiple sets of parameters that visually fit the SAXS patterns equally well when a PRISM-based structure factor is applied. This is demonstrated in Table S6 and Figure S4, which show SAXS pattern fits using a cylindrical form factor and PRISM-based structure factor and the corresponding fitting parameters for three different fixed cylinder lengths. Thus, the empirical structure factor used by the present study (eq 5) decouples β, *c*(*q*), and *P*_SC_(*q*) by treating β as a constant and substituting *P*_cyl_(*q*) for *P*_SC_(*q*). Implementation of this empirical structure factor is only intended to estimate the hard sphere interactions between scattering particles. To reduce the number of fitting parameters allowed to vary simultaneously, the SAXS patterns were first fit with eq S3 over *q* > *q*_max_. As the aggregate length fitting parameter was confirmed to be independent of the *P*_cyl_(*q*) fits for *q* > *q*_max_, the length parameter was fixed at 30 nm, the minimum persistence length estimated from SAS studies of dilute Nafion and C2 (Dow Chemical Co.) dispersions in aqueous solvents.^7,30^ Then, the fitting parameters of eq S3 were fixed before fitting the PFSA dispersion SAXS patterns over the entire *q* range (0.1 nm^–1^ ≤ *q* ≤ 2.5 nm^–1^) with eq 4. The fitting procedure sequence is demonstrated in Figure S5. The SAS model fits are plotted as solid lines against the experimental SAXS patterns in Figure 2 and are shown to be good fits to the data across the entire experimental *q*-range. Note that the stepwise fitting procedure was selected to minimize fitting uncertainties associated with key fitting parameters such as the aggregate radius and radius of closest approach. A single-step fitting process (where the form and structure factors are fit simultaneously) was found to be a slightly better fit to the data in the vicinity of *q*_max_ but resulted in higher fitting uncertainties of the aggregate radius and a loss of morphological distinction between different PFSA chemical structures and solvent compositions.

The aggregate radius values obtained from fitting the PFSA dispersion SAXS patterns in Figure 2a with eq 4 are shown in Table 2 and are comparable to results obtained from previous SAS studies of PFSA dispersions in aqueous and alcoholic solvents (about 2 nm).^5–7,21,30^ The radius values found by the present work are also close to cryo-TEM measurements done by Song et al. and Wang et al. on dilute C2 and LSC PFSA dispersions (∼1.5 to 1.8 nm).^22,38^ The aggregate radius can be observed to decrease as side chain length decreases or the side chain content increases (Table 2). The decrease in aggregate radius corresponds well with the decrease in average interaggregate spacing, where it was discussed earlier in the present work that radius generally decreases as side chain length decreases (at fixed side chain content) or side chain content increases (at fixed side chain length). This general relationship between aggregate radius and PFSA chemical structure was maintained at lower PFSA concentrations. The correlation between aggregate radius and average interaggregate spacing found by the present work is also in agreement with the findings of previous SAS studies of solutions of charged polymers.^7,26,30,35,52,74^ The value of *R*_ca_ in *S*(*q*) (eq 5) is mathematically correlated to the position of the experimental *q*_max_. Thus, *R*_ca_ follows the same trend as the average interaggregate spacing, equal to 2π/*q*_max_, as a function of side chain length and side chain content. The agreement between the SAS model fitting parameters and *q*_max_ shows that the SAS model can successfully attribute variations in *q*_max_ to changes in aggregate radius and interaggregate spacing.

The Hayter–Penfold rescaled mean spherical approximation (RMSA) structure factor was explored as an alternative to the PRISM-based structure factor used in the present work. The main assumptions of the RMSA structure factor are that the scattering particles are charged and that interparticle interferences (which generate a scattering maximum) occur due to screened electrostatic repulsions between charged scattering particles.^75^ In contrast, PRISM-based structure factors do not explicitly assume that scattering particles are charged.^70^ Therefore, the RMSA structure factor was investigated to determine if fits to the dispersion SAXS patterns could be improved by explicitly accounting for the charge of the PFSA aggregates.

A low-*q* power law plus cylindrical form factor with and without radius polydispersity and RMSA structure factor was used to fit a 25 wt % PFSA dispersion SAXS pattern. The fitting details are described in the Supporting Information, with fitting equations described by eqs S5 and S6, fits shown in Figure S6, and fitting parameters summarized in Table S7. The parameters that were varied during the fitting optimization are the cylinder radius, radius polydispersity where applicable, radius of closest approach, and charge of the scattering particles. The cylinder length fitting parameter was fixed at 5 nm, as the cylindrical form factor with RMSA structure factor was a poor fit to the scattering pattern for cylinder lengths >5 nm. Furthermore, the fitting uncertainty in the cylinder length was >100% when the cylinder length was allowed to vary as a fitting parameter along with the radius, radius of closest approach, and charge. Figure S6 shows that the cylindrical form factor with RMSA structure factor, both with and without radius polydispersity, is a reasonable fit to the dispersion SAXS pattern. Table S7 shows that the cylinder radius and radius of closest approach obtained by fitting with the RMSA model are 1.2 ± 0.4 and 1.8 ± 0.6 nm, respectively. The fit and fitting uncertainties were not improved by accounting for radius polydispersity with a Gaussian, log-normal, or Schulz distribution. These values from fitting with the RMSA structure factor are the same within error as the radius (1.16 ± 0.06 nm) and radius of closest approach (1.75 ± 0.01) obtained by fitting with the PRISM-based model.

Table S7 additionally shows that the scattering particle charge is 5 e with a fitting uncertainty of 80%. The RMSA structure factor was initially developed to describe the structure of macroions, i.e., systems with high charge density such as polyelectrolytes or small molecule micelles.^75,76^ In contrast, PFSAs are classified as ionomers due to their relatively low charge density of < ∼20 mol % charged groups (Table 1).^77^ Additionally, it is likely that not all PFSA side chains are located at the solvent–aggregate interface, resulting in a reduction of any effective surface charge on the aggregates.^78^ The combination of the inherent low charge functionality of PFSAs and relatively low surface charge of the PFSA aggregates contributes to an overall low charge density in semidilute PFSA dispersions. Thus, the RMSA structure factor does not appear to suitably quantify the effect of aggregate charge on the spatial ordering of PFSA aggregates. However, a different estimation of aggregate charge is discussed later in the present work.

The effect of the scattering maximum on the form factor fit and resulting aggregate radius values was further investigated by fully excluding the scattering maximum when fitting with the cylindrical form factor. In this modified approach, the form factor was fit to the data over *q* ≥ 2π/(*d* – ξ). This *q*-range corresponds to length scales smaller than the correlation length, where the scattered intensity is entirely unaffected by interparticle interferences. The cylindrical radius values obtained from the fits were similar to those obtained from the original fitting procedure and to previous reports (around 1 to 2 nm),^5–7,21,23,30,39^ but the fitting uncertainties were higher. The total fit to the data using the modified fitting approach is demonstrated in Figure S7 and indicates that the fit is poor relative to the original fitting approach where the form factor is fit over *q* > *q*_max_. Additionally, the modified fitting procedure resulted in an unexpected trend where the radius increased somewhat as the alcohol content of the solvent increased. This trend does not follow any previous investigations of PFSA aggregate dimensions using a variety of characterization techniques including SAS, DLS, and simulations.^31,36,78,79^ It should be noted that Yamaguchi et al. and Gupit et al. excluded the structure factor from their form factor fits to SAS patterns of more dilute PFSA dispersions.^35,36^ However, the scattering maximum in the SAXS patterns shown in Figure 2 occurs at significantly higher *q* (*q* > 0.9 nm^–1^) than the studies of Yamaguchi et al. and Gupit et al. due to the relatively high PFSA concentrations used in the present study. Thus, the unexpected trend where aggregate radius increases with increasing alcohol content of the solvent is likely due to the very limited *q*-range and reduced number of data points used in the modified form factor fit to the SAXS patterns in the present work. A more accurate form factor fit would likely require SAXS measurements including *q*-values as high as 4 nm^–1^, as in the work of Yamaguchi et al.^36^

The aggregate radius values obtained from the empirical SAS model developed by the present study may be used to further comment on PFSA aggregate morphology. By applying thermodynamic concepts developed to describe the self-assembly of micelles,^41^ Aldebert et al.^6^ postulated that the cylindrical radius of PFSA aggregates is determined by the balance between the solvent–aggregate interfacial energy, the elastic energy of the chains comprising the aggregate, and the energy associated with intrachain electrostatic repulsion between side chains.^80,81^ By assuming the latter is small compared to the elastic energy and interfacial energy, Aldebert et al.^6^ were able to investigate the influence of the elastic and interfacial energies on the aggregate radius by developing the model described by eqs 6 through 8. Equation 6 is the calculation of the aggregate surface area associated with one side chain, σ, where *V* is the aggregate volume normalized by EW, *R* is the aggregate radius, EW is the ionomer equivalent weight, *N*_A_ is Avogadro’s number, and ρ ≈ 2.1 g/cm^3^ is the PFSA density. Equation 7 describes the total energy of the aggregate normalized by EW, *E*_total_, equal to the sum of the elastic and interfacial energies, *E*_elastic_ and *E*_interfacial_, respectively, where *k* is the Boltzmann constant, *T* is the temperature, λ is the free end-to-end distance of a Gaussian chain, and γ is the solvent–aggregate interfacial energy. The elastic energy term can be interpreted as the energy required to form a PFSA aggregate with a core of hydrophobic backbone chains and polar side chains located at the aggregate surface. Minimization of *E*_total_ in eq 7 as a function of σ results in the relationship between σ and *E*_elastic_ and *E*_interfacial_ described by eq 8, where ${\left(\frac{2kTV^{2}}{\lambda^{2}}\right)}^{1/3}$ is the contribution of *E*_elastic_ and $\gamma^{-1/3}$ is the contribution of *E*_interfacial_ to the equilibrium value of σ, σ_e_.

6 $$\sigma =\frac{2V}{R},\mathrm{where}\;V=\frac{EW}{N_{A}\rho }$$

7 $$E_{total}=E_{elastic}+E_{\mathrm{int}\mathrm{erf}acial}=kT\frac{V^{2}}{\sigma^{2}\lambda^{2}}+\sigma \gamma$$

8 $$\sigma_{e}={\left(2kT\frac{V^{2}}{\lambda^{2}}\right)}^{1/3}\gamma^{-1/3}$$

The model of Aldebert et al. (eqs 6 through 8)^6^ was applied to the PFSA dispersions examined in this study. The aggregate surface area per side chain, σ, was calculated from the PFSA EW and radius values obtained from fitting the dispersion SAXS patterns with eq S3. The values of σ are listed in Table 2 as a function of PFSA chemical structure and show that the highest value of σ is found for the shortest side chain chemical structure. Assuming constant solvent–aggregate interfacial energy, λ, for a given solvent composition, eq 8 indicates that the equilibrium aggregate surface area per side chain, σ_e_, increases with increasing elastic energy of the side chains at the surface of the aggregate. Thus, a shorter side chain chemical structure may be associated with higher elastic energy. This is consistent with the predicted lower flexibility of a short, perfluorinated chain terminating in a polar sulfonate group. Comparison of the series of C4 ionomers with the same side chain chemical structure but different side chain contents (Table 1) shows that σ_e_ does not change significantly with side chain content or EW within error. However, eq 8 shows that σ_e_ is proportional to *V* ^2/3^ and thus EW^2/3^, which is a relatively weak dependence of σ_e_ on EW.^6^

The values of σ obtained from the model of Aldebert et al.^6^ may be validated by calculating the side chain length represented by λ in eq 8. This can be accomplished by using σ obtained from eq 6 and estimating the solvent–aggregate interfacial energy, γ, as 25.2 mJ/m^2^ from the interfacial energy between air and 50 wt % nPrOH (balance water) at room temperature.^82^ The calculated values of λ are summarized in Table 2 as a function of PFSA chemical structure and range from about 0.4 to 0.6 nm. These values appear to be in good agreement with PFSA side chain lengths obtained from simulations: about 0.5 nm for the C2 side chain chemical structure and about 0.7 nm for the C4 and LSC side chain chemical structures.^83,84^

The effect of solvent composition on PFSA aggregate dimensions was further investigated by calculating the surface area per side chain, σ, as a function of solvent composition. The σ values were calculated using eq 6, with *R* obtained from fitting the SAXS patterns in Figure 2b–d with eq S3. The fitting parameters are summarized in Table S8. A plot of σ as a function of [alcohol], shown in Figure 5a, indicates that σ increases (and aggregate radius decreases) with increasing [alcohol]. An assumption made to calculate σ (eq 6) is that all side chains are located at the solvent–aggregate interface.^6,30^ However, the work of Berlinger et al. relating the pH of PFSA dispersions to aggregate morphology demonstrated that a percentage of side chains could be located in the aggregate interior, away from the solvent–aggregate interface. This percentage of side chains was shown to depend on PFSA concentration and binary nPrOH–water solvent composition at ≤1 wt % PFSA. At 4 wt % PFSA, the percentage of side chains in the aggregate interior appeared to be independent of solvent composition.^78^ Thus, it can be assumed that the number of side chains at the solvent–aggregate interface in nondilute PFSA dispersions may (1) be lower than the total number of side chains in the system and (2) independent of solvent composition. These assumptions indicate that values of σ obtained from eq 6 are likely underestimates and any changes in σ are primarily due to changes in aggregate radius.

**Figure 5:**
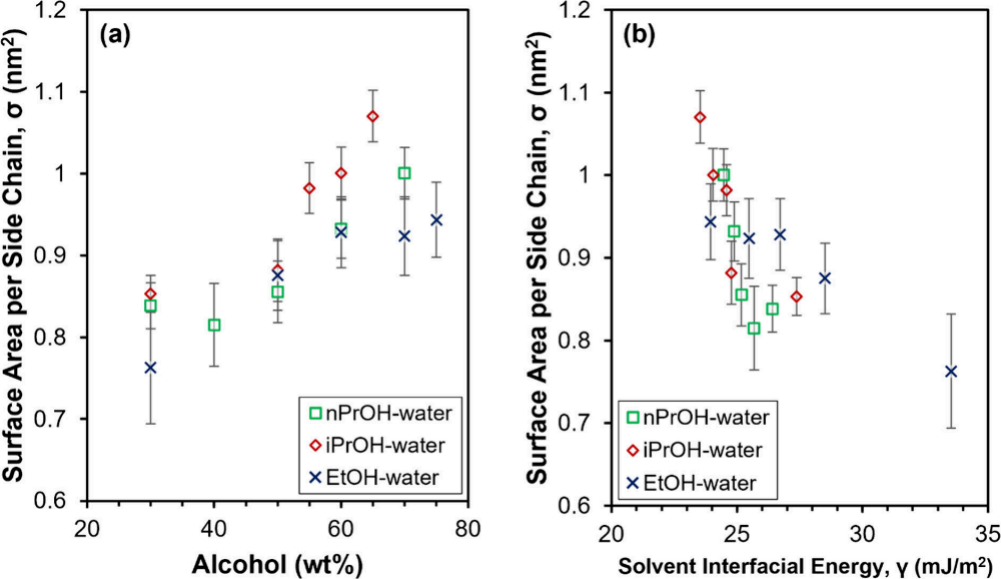
Aggregate surface area per side chain, σ, of 25 wt % 790 EW C4 dispersions in alcohol–water as a function of (a) alcohol concentration in the solvent (balance water) and (b) mixed alcohol–water solvent interfacial energy.

Changes in aggregate radius and thereby changes in σ may be affected by PFSA–solvent interactions and the average number of PFSA chains per aggregate, i.e., the aggregation number. Loppinet et al. showed that increases in PFSA aggregate radius reduced the total surface to volume ratio of the aggregates, thereby minimizing the solvent–aggregate interfacial energy.^30^ While the total surface to volume ratio of PFSA aggregates could not be quantified by the present work due to the limited *q*-range of the dispersion SAXS measurements, the surface area per side chain, σ, may be used to estimate the relative surface area of aggregates exposed to solvent. A plot of σ as a function of the estimated interfacial energy between the binary alcohol–water solvent system and the aggregates, approximated as the interfacial energy between the solvent and air,^82^ is shown in Figure 5b. It can be seen that σ increases with decreasing solvent interfacial energy, in qualitative agreement with the model of Aldebert et al.^6^ Additionally, an increase in σ suggests an improvement in the relative solvation of the PFSA aggregates. Thus, the broadening of *q*_max_ and decrease in spatial order observed in Figure 2b–d may be associated with the decrease in the interfacial solvent–aggregate energy at higher [alcohol]. These findings of the dependence of PFSA aggregate morphology on the alcohol–water solvent composition are also in agreement with other studies of PFSA dispersions, which suggest that improved compatibility between the solvent and hydrophobic component of the PFSA aggregates as [alcohol] increases results in less aggregation.^10,31,33,85,86^ The average number of PFSA chains per aggregate can be qualitatively evaluated using the aggregation number of a cylindrical micelle. The aggregation number is equal to π*R* ^2^ *L*/ν*V* = 2π*RL*/νσ, where *R* is the micelle radius, *L* is the length, ν is the number of side chains per PFSA chain, and σ is the surface area per polar headgroup, analogous to the surface area per side chain.^41^ It can be seen that the aggregation number is proportional to radius and inversely proportional to σ. Thus, the increase in σ with increasing [alcohol] may be attributed to a decrease in aggregate radius concurrent with a decrease in aggregation number. This further supports a reduction in aggregation as [alcohol] increases, where a larger number of smaller aggregates is expected. Furthermore, if the total number of side chains at the solvent–aggregate interface is mostly independent of solvent composition,^78^ then σ may be interpreted as an increase in exposure of the hydrophobic component of the PFSA aggregates to the solvent.

The side chains at the solvent–aggregate interface may also affect the spatial ordering of PFSA aggregates through electrostatic interactions. For example, Loppinet et al. found that the scattering maximum of PFSA dispersions broadened, i.e., the correlation length decreased, as the solvent dielectric constant decreased or as the concentration of excess salt increased, consistent with a reduction of interaggregate electrostatic interactions.^30^ Thus, spatial ordering or the correlation length is expected to decrease with decreasing number of side chains at the solvent–aggregate interface. The number of side chains at the solvent–aggregate interface is equal to the total interfacial surface area divided by the surface area per side chain, σ.^41^ For cylindrical micelles, the total interfacial surface area is equal to $\frac{2\phi }{R}\pi R^{2}L$ , where ϕ is the PFSA volume fraction, *R* is the aggregate radius, and *L* is the aggregate length.^87^ To avoid an assumption of aggregate length, the number of side chains per unit length, *N*_sc_/*L*, was calculated using the equation $\frac{N_{sc}}{L}=\frac{2\pi \phi R}{\sigma }$ . The values of *N*_sc_/*L* for the five different PFSA chemical structures and the 790 EW C4 in different alcohol–water solvent systems are plotted as a function of correlation length in Figure 6. It can be seen that *N*_sc_/*L* decreases as correlation length decreases, which is consistent with a reduction in interaggregate electrostatic interactions due to the polar side chains. Thus, the aggregation behavior and morphology of PFSAs in alcohol–water solvent systems appear to be affected by both ionomer–solvent interactions and interaggregate electrostatic interactions.

**Figure 6:**
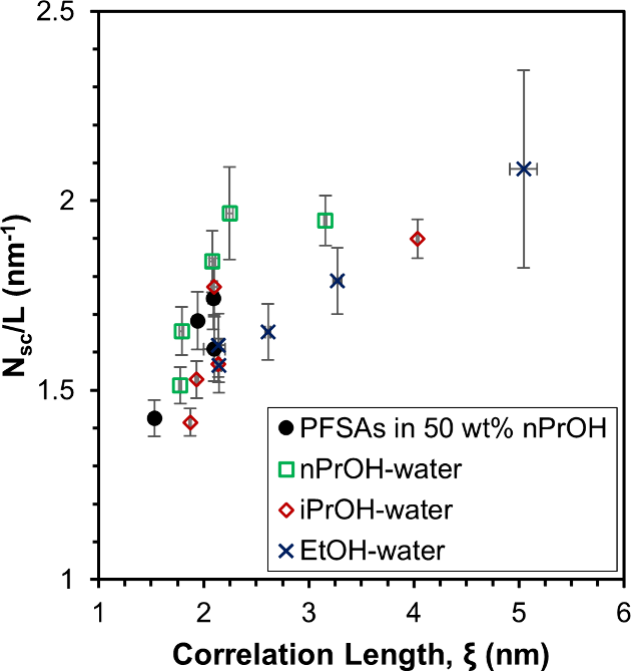
Number of side chains per unit length at the solvent–aggregate interface, *N*_sc_/*L*, as a function of correlation length for 25 wt % PFSA dispersions. Data represented by green squares, red diamonds, and blue crosses were obtained from 25 wt % 790 EW C4 in different alcohol–water solvent compositions.

In summary, changes in binary alcohol–water solvent composition can have a profound effect on the morphology and aggregation of PFSA dispersions. As [alcohol] in the solvent increases, (1) the spatial order of PFSA aggregates is decreased, (2) the aggregate surface area per side chain, σ, increases, and (3) the aggregation number decreases. These three effects are consistent with improved compatibility between the solvent and the hydrophobic component of the PFSA aggregates at higher [alcohol].

## Conclusions

The aggregate morphology of semidilute, H^+^-form PFSA dispersions was investigated as a function of PFSA chemical structure and mixed alcohol–water solvent composition using SAXS. Investigation of the power law scaling relationship between PFSA concentration and *q*_max_ further supports the presence of rodlike aggregates for dispersions of the five different PFSA chemical structures examined by the present study. Due to interaggregate correlations, the length of the rodlike aggregates was demonstrated to be unquantifiable with SAS models. However, a semiempirical SAS model was developed by the present study to fully fit the PFSA dispersion SAXS patterns and quantify the aggregate radius and scattering maximum arising from interaggregate correlations. The aggregate radius values were used to calculate the aggregate surface area associated with one side chain, σ, using a model developed by Aldebert et al.^6^ The surface area per side chain was shown to depend on side chain length and solvent composition. Higher values of σ for shorter side chain chemical structures were attributed to lower flexibility expected for shorter side chains. Higher alcohol fractions in the solvent were also shown to result in higher σ and attributed to a decrease in solvent–aggregate interfacial energy. An increase in σ was interpreted as an increase in exposure of the hydrophobic component of the PFSA aggregates to the solvent. The present work has demonstrated for the first time that thermodynamic micellar self-assembly concepts such as σ can be used to describe the morphology of FPSA aggregates and can be applied to all commercially available PFSA chemistries and three different alcohol–water solvent systems. The second part of our study will demonstrate a strong relationship between σ obtained from SAXS measurements of PFSA dispersions and zero-shear viscosity of the same dispersions, establishing a connection between semidilute PFSA dispersion morphology and rheological properties.

## Supplementary Material



## Notes

Additional information on and fitting parameters of SAXS pattern fits to various models, additional SAXS patterns, and SAXS pattern vertical shift factors (PDF)

## §

Johnson Matthey Technology Centre, Blounts Court, Sonning Common, Reading, RG4 9NH, United Kingdom

## Abbreviations

cryo-TEM: cryogenic transmission electron microscopy
DLS: dynamic light scattering
EtOH: ethanol
EW: equivalent weight
iPrOH: isopropanol
nPrOH: n-propanol
PFSA: perfluorosulfonic acid ionomer
PRISM: Polymer Reference Interaction Site Model
RMSA: rescaled mean spherical approximation
SAS: small-angle scattering
SAXS: small-angle X-ray scattering
TFE: tetrafluoroethylene
