# Supplementary material for: Effect of Ionomer–Solvent Interactions in PFSA Dispersions: Dispersion Morphology
Source: Macromolecules. 2025 Aug 6;58(16):8854–65. doi: 10.1021/acs.macromol.5c00613 (PMC12392727; doi:10.1021/acs.macromol.5c00613)
Supplement: Supplementary file 1 [file ma5c00613_si_001.pdf]

SUPPORTING INFORMATION

EFFECT OF IONOMER-SOLVENT INTERACTIONS IN PFSA DISPERSIONS: DISPERSION MORPHOLOGY

Melissa Novy [a†], Denis Duchesne [b†], Gregg Dahlke [b], Lisa P. Chen [b], and Robert B. Moore [a]*

* *Email: rbmoore3@vt.edu*

[a] Macromolecules Innovation Institute, Department of Chemistry, Virginia Tech, Blacksburg, Virginia, 24061, United States.

[b] 3M Advanced Materials Division, 3M Center, Building 280-1W-03, St. Paul, Minnesota 55144, United States.

Present Address

†Johnson Matthey Technology Centre, Blounts Court, Sonning Common, Reading, RG4 9NH, United Kingdom.

Vertical Shifting Factors of PFSA Dispersion SAXS Patterns

Table S1. Vertical shifting factors of SAXS patterns of 25 wt% PFSA in 50 wt% nPrOH shown in **Figure 2a**.

PFSA	Shift
830 EW C2	1
725 EW C4	20
790 EW C4	60
910 EW C4	100
940 EW LSC	290

Table S2. Vertical shifting factors of SAXS patterns of 25 wt% 790 EW C4 in different alcohol-water solvent compositions shown in **Figure 2b-d**.

Solvent Composition	Alcohol (wt%)	Shift
nPrOH-water	30	3600
	40	150
	50	40
	60	7
	70	1
iPrOH-water	30	1000
	50	400
	55	50
	60	10
	65	1
EtOH-water	30	2000

	50	220
	60	35
	70	6
	75	1

Effect of Side Chain Chemical Structure on Copolymer Composition

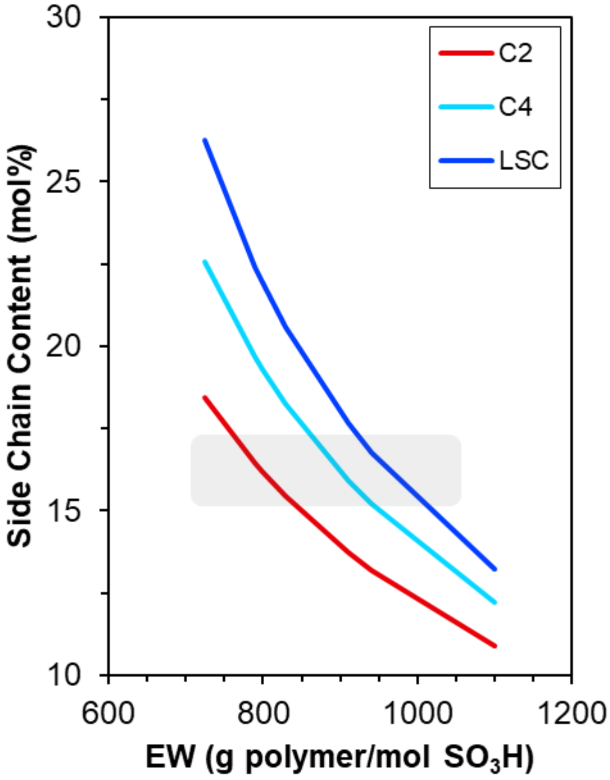

Figure S1. PFSA side chain content as a function of EW for the three different side chain chemical structures. The gray box indicates that the side chain content is similar for the 830 EW C2, 910 EW C4, and 940 EW LSC PFSAs.

Fitting SAXS Patterns of PFSA Dispersions with the Teubner-Strey Model

To quantify the shape and position of q_{max} without assuming aggregate form or interactions, the SAXS patterns of PFSA dispersions were fit with the sum of a low-q power law and the Teubner-

Strey model that takes the form shown in **eq S1**. The first and last terms in **eq S1** are the power law representing the low-q upturn, where A_{T1} is a scaling factor and D is the slope of the power law, and a constant background, B, respectively. The second term is the Teubner-Strey equation representing the scattering maximum, where A_{T2} is the scaling factor of the second term, q_{max} is the position of the scattering maximum, and ξ is the dispersion in d-spacing, i.e., the correlation length. The correlation length may be interpreted as the degree of spatial order in the PFSA dispersions, where small correlation lengths may indicate low spatial order. The fitting parameters of **eq S1** as a function of PFSA chemical structure are summarized in **Table S3**. **Table S4** shows the fitting parameters as a function of solvent composition for 25 wt% 790 EW C4 dispersions.

$$I(q) = \frac{A_{T1}}{q^D} + A_{T2}\phi\frac{8\pi}{\xi}\left(\frac{a}{c_2} + \frac{c_1}{c_2}q^2 + q^4\right)^{-1} + B \qquad (S1)$$

$$\text{where } \frac{a}{c_2} = [1 + (q_{max}\xi)^2]^2\xi^{-4}$$

$$\text{and } \frac{c_1}{c_2} = [-2\xi^2(q_{max}\xi)^2 + 2\xi^2]\xi^{-4}$$

Table S3. Teubner-Strey model (**eq S1**) fitting parameters as a function of PFSA chemical structure at 25 wt% PFSA in 50 wt% nPrOH (balance water).

PFSA	Scattering Maximum, q_{max} (nm^{-1})	Average Inter-aggregate Distance (nm)	Correlation Length, ξ (nm)
830 EW C2	1.050 ± 0.004	5.98 ± 0.02	1.53 ± 0.02
725 EW C4	1.31 ± 0.01	4.81 ± 0.03	2.09 ± 0.05
790 EW C4	1.17 ± 0.01	5.36 ± 0.03	2.08 ± 0.06
910 EW C4	0.945 ± 0.003	6.65 ± 0.02	1.94 ± 0.02
940 EW LSC	0.93 ± 0.01	6.8 ± 0.1	2.1 ± 0.1

Table S4. Teubner-Strey model (**eq S1**) fitting parameters as a function of solvent composition for 25 wt% 790 EW C4 dispersions in three different alcohol-water systems.

Solvent Composition	Alcohol (wt%)	Scattering Maximum, q_{max} (nm^{-1})	Average Inter-aggregate Distance (nm)	Correlation Length, ξ (nm)
nPrOH-water	30	1.02	6.15	3.16
	40	1.18	5.32	2.32
	50	1.17	5.36	2.08
	60	1.11	5.65	1.89
	70	1.06	5.90	1.82
EtOH-water	30	0.89	7.03	5.05
	50	1.02	6.18	3.27
	60	1.02	6.14	2.61
	70	1.03	6.07	2.14
	75	0.96	6.53	2.14
iPrOH-water	30	1.05	5.99	4.04
	50	1.04	6.05	2.10
	55	1.11	5.64	2.14
	60	1.13	5.56	1.93
	65	1.06	5.91	1.87

Fitting SAXS Patterns with Different Form Factors

Equations S2 through **S4** describe the theoretical scattered intensities of a spherical form factor, $P_{sphere}(q)$, cylindrical form factor with finite length, $P_{cyl}(q)$, and cylindrical form factor with infinite length, $P_{cyl\infty}(q)$,[1,2] respectively. The independent parameters common to these form factors are

scale (i.e., a constant that shifts the scattering pattern vertically), R, the radius, and BG, the incoherent scattering background. The cylindrical form factors (**eqs S3** and **S4**) have two additional independent parameters: L, the cylinder length, and α, the angle between the scattering vector and cylinder axis. A spherical form factor and three different cylindrical form factors with fixed cylinder lengths of 10 nm, 30 nm and infinity are plotted along with an experimental 25 wt% PFSA dispersion SAXS pattern in **Figure S2**.

$$P_{sphere}(q) = \frac{scale}{\frac{4}{3}\pi R^3}\left[4\pi R^3 \frac{\sin qR - qR\cos qR}{(qR)^3}\right]^2 + BG \qquad (S2)$$

$$P_{cyl}(q,\alpha) = \frac{scale}{\pi R^2 L}\int_0^{\frac{\pi}{2}} F^2(q,\alpha)\sin\alpha\, d\alpha + BG \qquad (S3)$$

where $F(q,\alpha) = 2\pi R^2 L \frac{\sin\left(\frac{1}{2}qL\cos\alpha\right)}{\frac{1}{2}qL\cos\alpha}\frac{J_1(qR\sin\alpha)}{qR\sin\alpha}$ and $J_1(x)$ is the first-order Bessel function

$$P_{cyl\infty}(q) = scale \times \frac{R^2}{2q}\left(2\frac{J_1(qR)}{qR}\right)^2 + BG \qquad (S4)$$

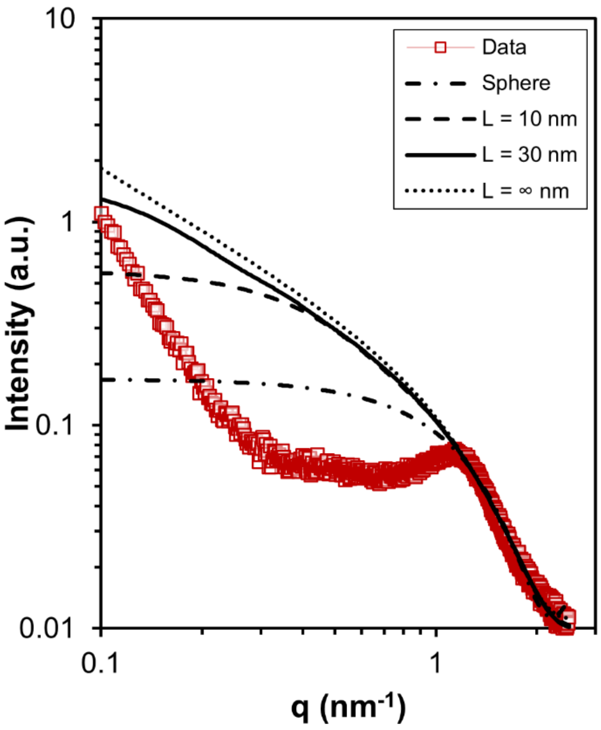

Figure S2. Comparison of fitting dispersion SAXS data of 25 wt% 790 EW C4 in 50 wt% nPrOH (balance water) with different form factors: spherical and cylindrical with fixed cylinder lengths, L, of 10, 30, and ∞ nm over $q > q_{max}$.

Table S5. Radius fitting parameters from fitting over $q > q_{max}$ with different form factors.

Form Factor	Radius (nm)
Sphere	1.30 ± 0.01
Cylinder, L = 10 nm	1.52 ± 0.5
Cylinder, L = 30 nm	1.49 ± 0.4
Cylinder, L = ∞ nm	1.54 ± 0.01

SAXS Patterns of Different PFSAs in 50 wt% nPrOH

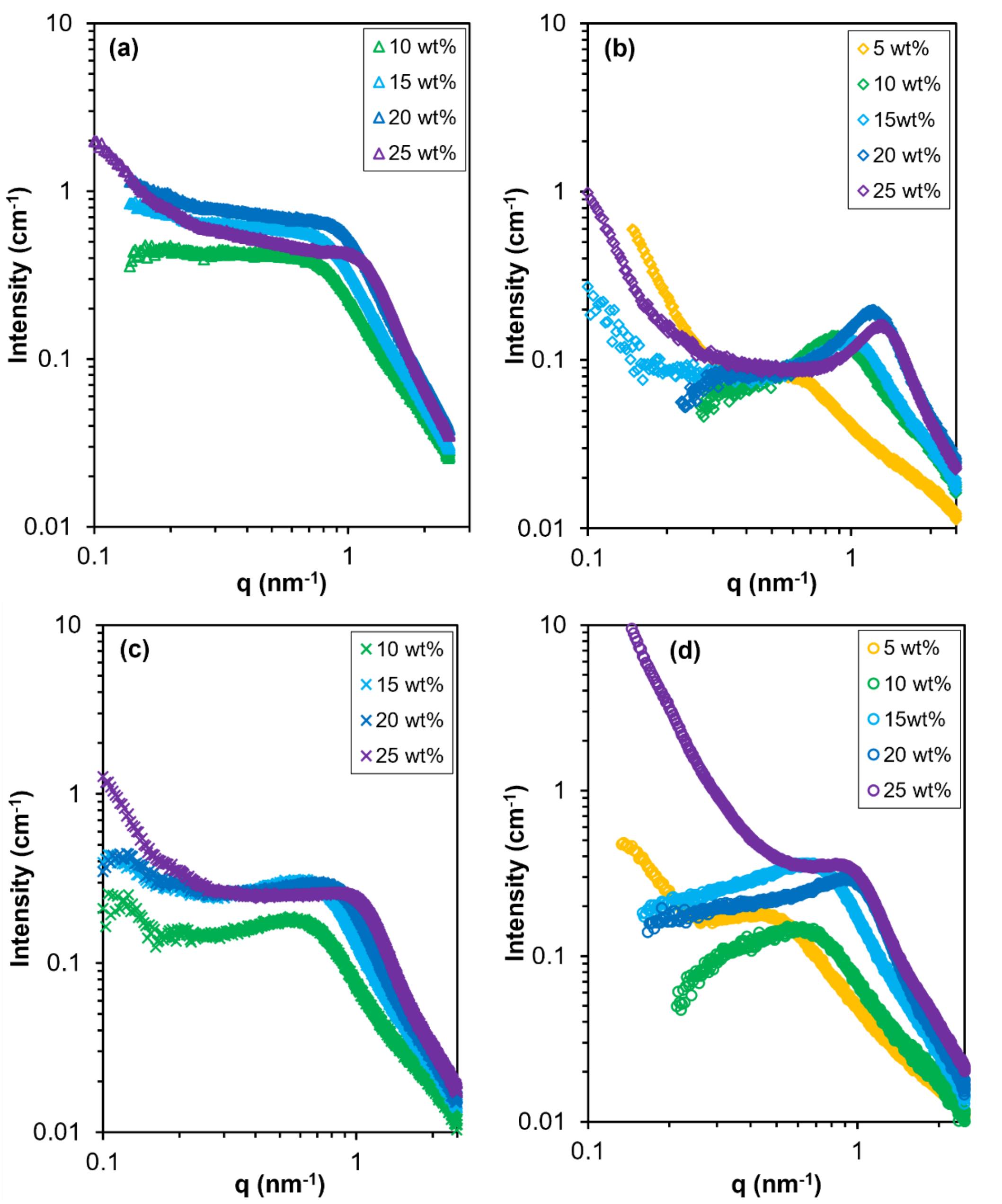

Figure S3. Dispersion SAXS patterns in 50 wt% nPrOH as a function of PFSA concentration of (a) 830 EW C2, (b) 725 EW C4, (c) 910 EW C4, and (d) 940 EW LSC.

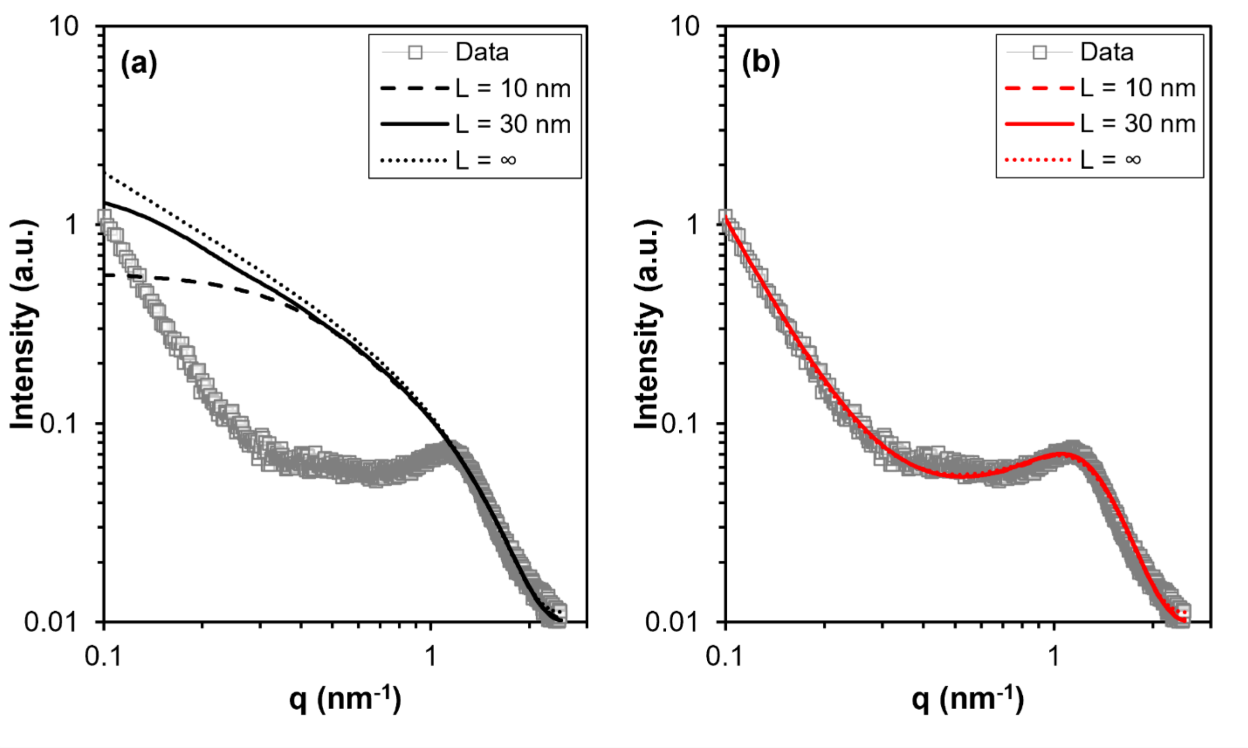

Figure S4. Demonstration of fitting the SAXS pattern of 25 wt% 790 EW C4 in 50 wt% nPrOH (balance water) with (a) a cylindrical form factor with fixed cylinder lengths, L, of 10, 30, and ∞ nm over the range $q > q_{max}$ and (b) a cylindrical form factor with PRISM-based structure factor and low-q power law over the range $0.1 < q < 2.5$ nm^{-1}, with the same fixed L values of 10, 30, and ∞ nm.

Table S6. Fitting parameters of the cylindrical form factor with a PRISM-based structure factor and low-q power law as a function of fixed cylinder length.

Cylindrical Form Factor		PRISM-Based Structure Factor		Low-q Power Law	
Cylinder Length (nm)	Radius, R (nm)	Radius of Closest Approach, R_{ca} (nm)	β	Scale × 10^{-3}	Power Law Exponent
10	1.52 ± 0.46	1.75 ± 0.01	25.1 ± 0.5	1.00 ± 0.02	3.02 ± 0.01
30	1.49 ± 0.43	1.77 ± 0.01	25.5 ± 0.5	1.00 ± 0.02	3.02 ± 0.01
∞	1.54 ± 0.01	1.69 ± 0.01	23.4 + 0.5	0.07 ± 0.02	3.17 ± 0.01

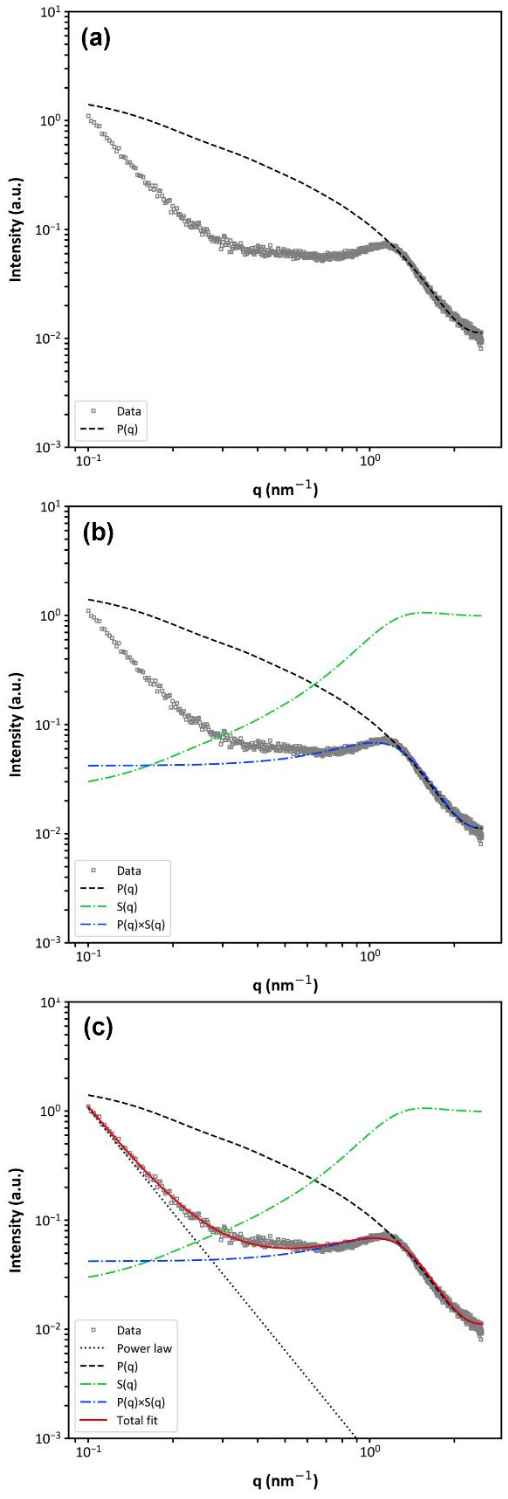

Figure S5. Example procedure for fitting dispersion SAXS patterns with **eq 4**. The example SAXS pattern data is of a 25 wt% 790 EW C4 dispersion in 50 wt% nPrOH (balance water). (a) Cylindrical form factor, P(q), fit to the data over $q > q_{max}$, with fixed cylinder length of 30 nm

(black dashed line); (b) empirical structure factor, S(q) (green dot-dashed line), and P(q)×S(q) (blue dot-dashed line); (c) low-q power law fit (black dotted line) and total fit to the data (red line) over the entire q-range ($0.1\ nm^{-1} \leq q \leq 2.5\ nm^{-1}$).

Fitting SAXS Patterns with a Low-q Power Law Plus Cylindrical Form Factor and Hayter-Penfold Rescaled Mean Spherical Approximation Structure Factor

A 25 wt% PFSA dispersion SAXS pattern was fit with a low-q power law plus cylindrical form factor and Hayter-Penfold rescaled mean spherical approximation (RMSA) structure factor in SasView. The general form of the fitting equation is described by **eq S5**, where A_1, A_2, and A_3 are scaling factors, D is the slope of the power law, and B is a constant background. The Hayter-Penfold RMSA structure factor is given by **eq S6**. The values of the function a(K) are described in the work of Hayter and Penfold[3] and c is the volume fraction of scattering particles. The fits are shown in **Figure S6**.

$$I(q) = \frac{A_1}{q^D} + A_2 \mathrm{P_{cyl}}(q) \times \mathrm{A_3 S_{RMSA}(q)} + B \qquad (S5)$$

$$S_{RMSA}(K) = \frac{1}{1 - 24ca(K)} \qquad (S6)$$

Table S7. Fitting parameters of the low-q power law plus cylindrical form factor and Hayter-Penfold RMSA structure factor.

Fitting Parameter	Monodisperse Radius Fit	Polydisperse Radius Fit
Radius (nm)	1.2 ± 0.4	1.2 ± 0.6
Radius Polydispersity (Ratio of Standard Deviation to Average Radius)	N/A	0.2 ± 0.7

Radius of Closest Approach (nm)	1.8 ± 0.6	1.8 ± 0.6
Charge (e)	5 ± 4	5 ± 4

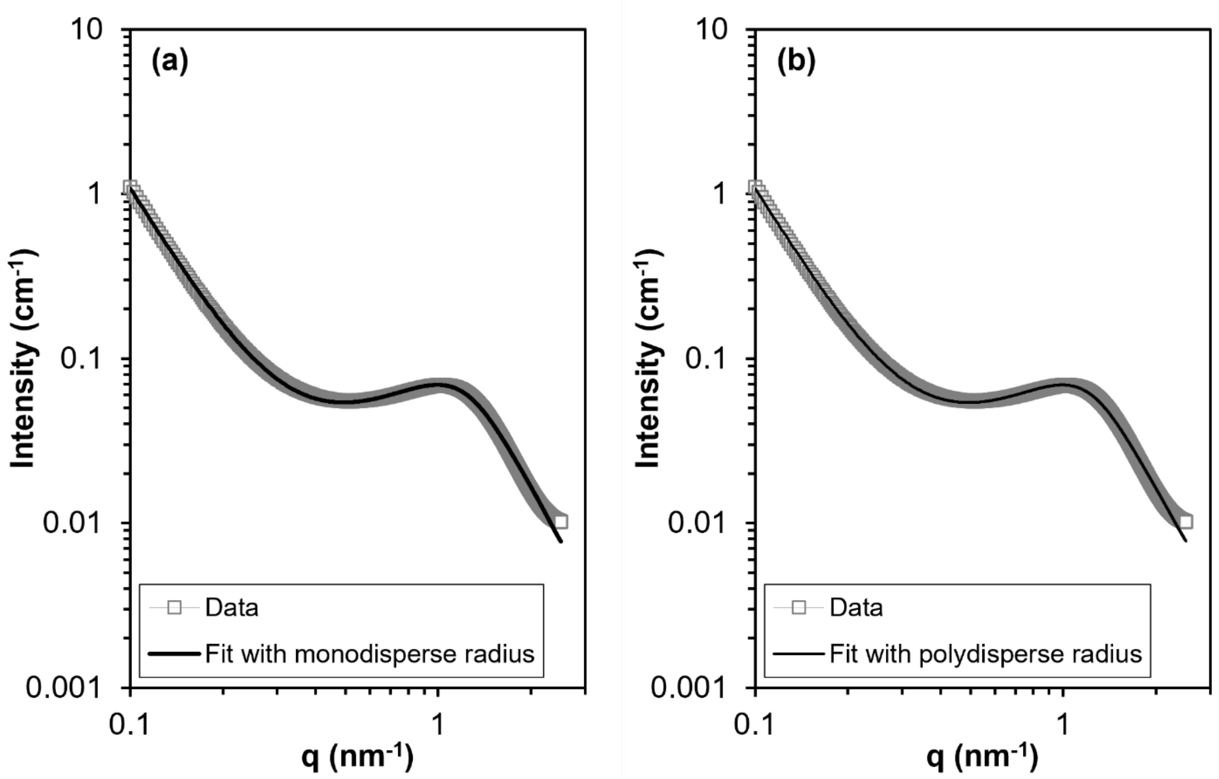

Figure S6. Low-q power law plus cylindrical form factor with RMSA structure factor (a) without radius polydispersity and (b) with radius polydispersity. Data is the SAXS pattern of the 25 wt% 790 EW C4 PFSA in 50 wt% nPrOH.

Fitting the Cylindrical Form Factor Over $q \geq 2\pi/(d - \xi)$

To further investigate the effect of the scattering maximum on the form factor fit, the empirical SAS model developed by the present work was modified to fit the form factor over $q \geq 2\pi/(d - \xi)$, corresponding to lengthscales entirely unaffected by interparticle interferences. The fitting components and total fit of this modified approach are shown in **Figure S7**.

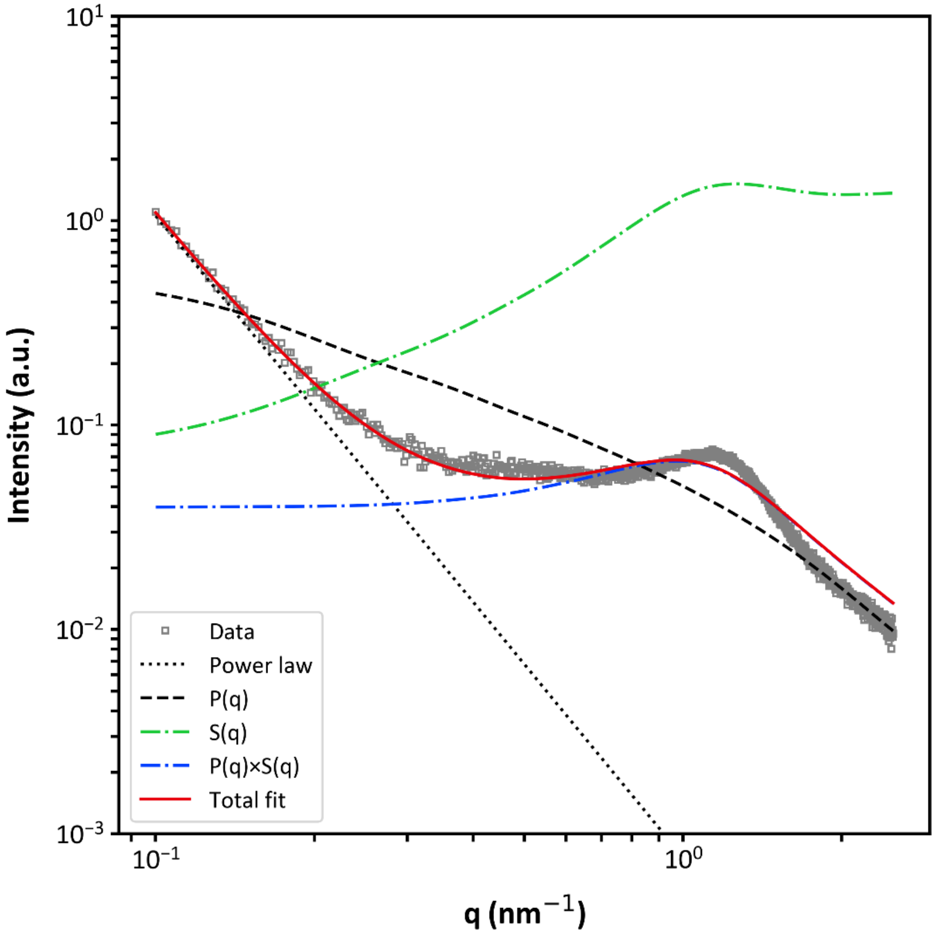

Figure S7. Example procedure for fitting dispersion SAXS patterns where the form factor is fit over $q \geq 2\pi/(d - \xi)$. The example SAXS pattern data is of a 25 wt% 790 EW C4 dispersion in 50 wt% nPrOH (balance water). The fitting components are a cylindrical form factor, P(q), fit to the data over $q \geq 2\pi/(d - \xi)$, with fixed cylinder length of 30 nm (black dashed line); empirical structure factor, S(q) (green dot-dashed line), and P(q)×S(q) (blue dot-dashed line); low-q power law fit (black dotted line) and total fit to the data (red line) over the entire q-range ($0.1\ nm^{-1} \leq q \leq 2.5\ nm^{-1}$).

Fitting Parameters of the Empirical SAS Model as a Function of Solvent Composition

Table S8. Aggregate radius, radius of closest approach, and surface area per side chain, σ, of 25 wt% 790 EW C4 dispersions at fixed cylinder length of 30 nm as a function of solvent composition for three different alcohol-water systems.

Solvent Composition	Alcohol (wt%)	Radius, R (nm)	Radius of Closest Approach, R_{ca} (nm)	Surface Area per Side Chain, σ (nm^2)
nPrOH-water	30	1.49 ± 0.05	2.46 ± 0.02	0.84 ± 0.03
	40	1.5 ± 0.1	1.77 ± 0.01	0.82 ± 0.05
	50	1.46 ± 0.06	1.75 ± 0.01	0.86 ± 0.04
	60	1.34 ± 0.05	1.92 ± 0.01	0.93 ± 0.04
	70	1.25 ± 0.04	2.14 ± 0.01	1.00 ± 0.03
iPrOH-water	30	1.46 ± 0.04	2.51 ± 0.01	0.85 ± 0.02
	50	1.42 ± 0.06	2.05 ± 0.01	0.88 ± 0.04
	55	1.27 ± 0.04	2.11 ± 0.01	0.98 ± 0.03
	60	1.25± 0.04	2.02 ± 0.01	1.00 ± 0.03
	65	1.17 ± 0.03	2.29 ± 0.01	1.07 ± 0.03
EtOH-water	30	1.6 ± 0.2	2.80 ± 0.02	0.76 ± 0.07
	50	1.43 ± 0.07	2.39 ± 0.01	0.88 ± 0.04
	60	1.35 ± 0.06	2.28 ± 0.01	0.93 ± 0.04
	70	1.35 ± 0.07	2.03 ± 0.01	0.92 ± 0.05
	75	1.32 ± 0.06	2.28 ± 0.01	0.94 ± 0.05

References

(1) Loppinet, B.; Gebel, G. Rodlike Colloidal Structure of Short Pendant Chain Perfluorinated Ionomer Solutions. *Langmuir* **1998**, *14* (8), 1977–1983. https://doi.org/10.1021/la9710987.

(2) Guinier, A.; Fournet, G. *Small-Angle Scattering of X-Rays*; Structure of Matter Series; John Wiley & Sons, Inc.: New York, NY, 1955.

(3) Hayter, J. B.; Penfold, J. An Analytic Structure Factor for Macroion Solutions. *Molecular Physics* **1981**, *42* (1), 109–118.
